# Effects of Isorhamnetin on Adipocyte Mitochondrial Biogenesis and AMPK Activation

**DOI:** 10.3390/molecules23081853

**Published:** 2018-07-25

**Authors:** Mak-Soon Lee, Yangha Kim

**Affiliations:** Department of Nutritional Science and Food Management, Ewha Womans University, 52 Ewhayeodae-gil, Seodaemun-gu, Seoul 03760, Korea; troph@hanmail.net

**Keywords:** isorhamnetin, adipogenesis, mitochondrial biogenesis, AMPK, 3T3-L1 adipocytes

## Abstract

Isorhamnetin (ISOR), 3-*O*-methylquercetin, is a naturally occurring flavonoid in many plants. It is a metabolite derived from quercetin and is known to exert beneficial effects on the prevention of obesity. However, the molecular mechanism of action involved in ISOR-mediated mitochondrial biogenesis, and AMP-activated protein kinase (AMPK) activation in 3T3-L1 cells remains unclear. The aim of this study was to determine whether ISOR affected mitochondrial biogenesis and AMPK activation, during 3T3-L1 adipocyte differentiation. Intracellular lipid and triglyceride accumulation, and glycerol-3-phosphate dehydrogenase (GPDH) activity decreased in ISOR-treated cells. The mRNA levels of adipogenic genes, such as the proliferator-activated receptor-γ (PPAR-γ), and adipocyte protein 2 (aP2), were inhibited by ISOR. In contrast, mRNA levels of mitochondrial genes, such as peroxisome proliferator-activated reporter gamma coactivator-1α (PGC-1α), nuclear respiratory factor (NRF)-1, transcription factor A (Tfam), and carnitine palmitoyl transferase-1α (CPT-1α), were all stimulated by ISOR treatment. Mitochondria DNA (mtDNA) copy number and AMPK activity were also stimulated by ISOR. The results suggested that the mitochondrial biogenic effect of ISOR in adipocytes might have been associated with stimulation of mitochondrial gene expression, mtDNA replication, and AMPK activation.

## 1. Introduction

Adipose tissue is an endocrine type of tissue that plays a central role in regulating energy homeostasis. Excessive accumulation of fat in adipose tissue causes obesity; and obesity-induced adipocyte mitochondria-dysfunction can substantially disrupt energy homeostasis. In particular, mitochondrial dysfunction in adipose tissue can lead to lipodystrophy syndrome, with metabolic and cardiovascular complications [1]. Mitochondrial dysfunction in various tissues is associated with the development of obesity and type-2 diabetes [2,3]. Wilson-Fritch et al. [4] observed a decrease in the levels of transcripts for nuclear-encoded mitochondrial genes accompanying the onset of obesity. The peroxisome proliferator-activated receptor γ coactivator-1α (PGC-1α) is considered the metabolic regulator of mitochondrial biogenesis [5]. PGC-1α regulates mitochondrial biogenesis and function through induction of the expression of nuclear respiratory factors, NRF-1 and NRF-2 [6,7]. Additionally, PGC-1α indirectly regulates the expression of mitochondria DNA (mtDNA) transcription by increasing the expression of mitochondrial transcription factor A (Tfam) [7,8]. Recently, there has been increasing interest in the regulation of mitochondrial function to prevent obesity.

**Figure 1:**
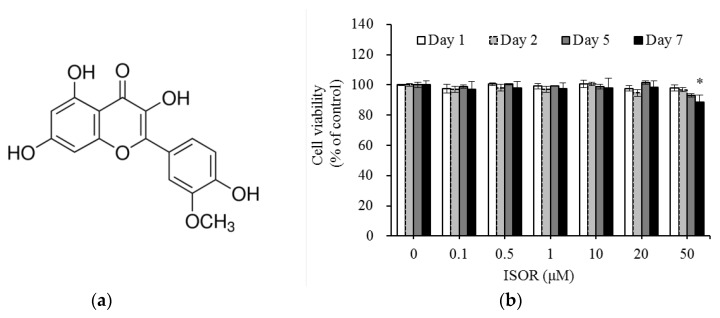
Chemical structures (**a**) and cell viability (**b**) of ISOR-treated 3T3-L1 cells. Cells were treated with 0 (control), 0.1, 0.5, 1, 10, 20, or 50 µM ISOR, and incubated for 1, 2, 5, or 7 days. Cell viability was determined using the water-soluble tetrazolium salt (WST)-8 assay. Values are expressed as means ± SE (*n* = 3) of three independent experiments. ISOR, isorhamnetin. * *p* < 0.05 vs. control.

Isorhamnetin (ISOR), known as 3-*O*-methylquercetin (Figure 1a), a metabolite of quercetin, is a naturally occurring flavonoid in many vegetables and fruits [9]. Quercetin exerted an anti-adipogenesis effect by inhibition of proliferator-activated receptor-γ (PPAR-γ), CCAAT/enhancer binding protein-α (CEBP-α), and activation of AMP-activated protein kinase (AMPK) signaling pathway in 3T3-L1 cells [10,11]. Dietary quercetin, ameliorated high fat diet-induced obesity by AMPKα1/SIRT1 activation [12]. Furthermore, quercetin has been shown to regulate the mitochondrial gene expression and mtDNA replication, in 3T3-L1 cells and diet-induced obese mice [13,14]. Indeed, when quercetin was orally administered to mice, it was quickly absorbed and reached the highest concentration in plasma at 1 h, though it remarkably decreased at 4 h. In contrast, the plasma concentration of ISOR, the major metabolite of quercetin, was increased rapidly and maintained longer than that of quercetin [15], which implied that quercetin was rapidly metabolized to ISOR after absorption in the body. ISOR has been reported to have various beneficial effects as an anti-oxidant [16], anti-inflammatory [17], anti-tumor [18], and anti-adipogenic [19] agent. A recent study reported that dietary ISOR is a PPARr antagonist that has protective effects against obesity and hepatic steatosis in diet-induced obese mice [20]. Lee and colleagues [19] indicated that ISOR inhibited adipogenesis through downregulation of PPAR-γ and CEBP-α in 3T3-L1 adipocytes. In addition, activated AMPK by ISOR showed a protective effect of hepatocyte mitochondrial dysfunction on the oxidative stress induced in HepG2 cells [21]. Nevertheless, it remains uncertain whether ISOR affects the adipocyte mitochondrial biogenesis, and AMPK activation in 3T3-L1 cells. 

This study, elucidates the effect of ISOR on mitochondrial biogenesis, and AMPK activation during 3T3-L1 adipocyte differentiation; furthermore, it identifies the molecular mechanisms underlying its regulatory action. 

## 2. Results 

### 2.1. Effect of ISOR on 3T3-L1 Cell Viability

ISOR was tested for the potential cytotoxic effects it might exert on 3T3-L1 cells. Cells were treated with ISOR at various concentrations (0 (control), 0.1, 0.5, 1, 10, 20, or 50 μM), and incubated for 1, 2, 5, or 7 days. There were no cytotoxic effects caused by ISOR at 0.1, 0.5, 1, 10, or 20 μM, after a 7-day incubation period (Figure 1b). However, incubation for 7 days in ISOR at 50 μM, significantly decreased cell viability by 11.4%, compared to the control treatment. Since cells did not show any toxicity upon treatment with ISOR at concentrations between 0.1 and 20 µM, this *in vitro* study was performed using ISOR at a nontoxic concentration range below 20 μM.

### 2.2. Effects of ISOR on Lipid and TG Accumulation in Adipocytes

**Figure 2:**
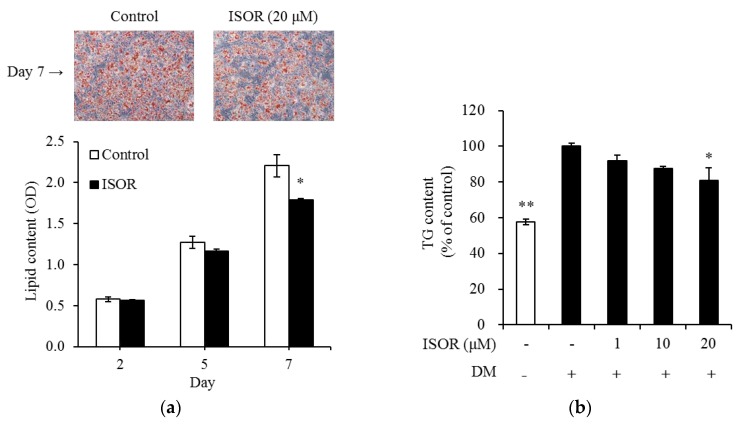
Effects of ISOR on intracellular lipid and TG accumulation during adipocyte differentiation. 3T3-L1 cells were treated with 0 (control) or 20 µM ISOR, and incubated for 2, 5, or 7 days (d2 to d9). On day 7 (d9), change of adipocyte differentiation was presented with Oil Red O-staining. Intracellular lipid content (**a**) was stained with oil-red O dye, the stained oil droplets were dissolved with isopropanol and quantified by spectrophotometry. Representative cell images were captured at 200× magnification. Intracellular TG content (**b**) was treated with 0 (DM-treated control), 1, 10, and 20 µM ISOR, and incubated for 7 days, and determined using enzymatic colorimetric methods. Values are expressed as means ± SE (*n* = 3) of three independent experiments. TG, triglyceride; ISOR, isorhamnetin; DM, differentiation medium containing 3-isobutyl-1-methylxanthine, dexamethasone and insulin. * *p* < 0.05 and ** *p* < 0.01 vs. untreated control.

The effects of ISOR on lipid and TG accumulation during adipogenesis were measured, and 3T3-L1 adipocytes were treated with 0 (control) or 20 μM ISOR, for 2, 5, or 7 days (d2 to d9). Intracellular lipid content was measured using Oil Red O-staining. On Day 7 (d9), change of adipocyte differentiation was observed with Oil Red O-staining (Figure 2a). After 7 days of incubation, ISOR inhibited the intracellular lipid content by 19.1%, compared to control cells (Figure 2a). To determine the intracellular TG content, 3T3-L1 adipocytes were treated with 0 (DM-treated control), 1, 10, or 20 μM ISOR for 7 days. The TG content significantly decreased by 19.3% in the presence of 20 μM ISOR, compared to the DM-treated control (Figure 2b).

### 2.3. Effect of ISOR on GPDH Activity in Adipocytes

**Figure 3:**
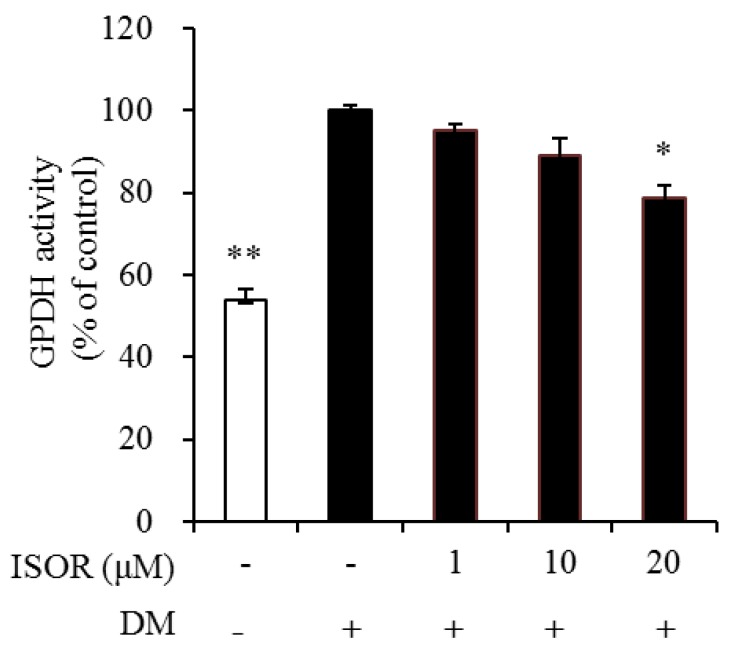
Effects of ISOR on GPDH activity in adipocytes. GPDH activity was determined using a GPDH assay kit. 3T3-L1 adipocytes were treated with 0 (DM-treated control), 1, 10, or 20 µM ISOR, and incubated for 7 days. Values are expressed as means ± SE (*n* = 3) of three independent experiments. GPDH, glycerol-3-phosphate dehydrogenase; ISOR, isorhamnetin; DM, differentiation medium containing 3-isobutyl-1-methylxanthine, dexamethasone, and insulin. * *p* < 0.05 and ** *p* < 0.01 vs. DM-treated control.

To elucidate the mechanism by which ISOR inhibits lipid and TG accumulation, GPDH activity was measured in ISOR-treated 3T3-L1 adipocytes. Cells were incubated with 0 (DM-treated control), 1, 10, and 20 μM ISOR, and incubated for 7 days. GPDH activity was significantly decreased by 21.5% in the presence of 20 μM ISOR, compared to the DM-treated control (Figure 3)

### 2.4. Effects of ISOR on the Expression of Genes Involved in Adipogenesis and Mitochondrial Function in Adipocytes

**Figure 4:**
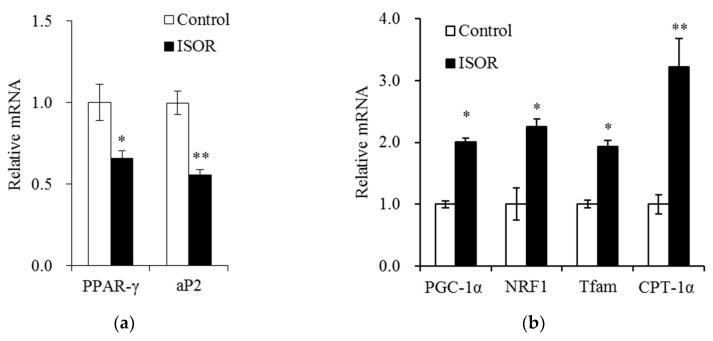
Effects of ISOR on the expression of genes involved in adipogenesis, and mitochondrial function in adipocytes. 3T3-L1 adipocytes were treated with 0 (control) or 20 μM ISOR, for 7 days. The mRNA levels were measured by qRT-PCR. Values are expressed as means ± SE (*n* = 3) of three independent experiments. ISOR, isorhamnetin. * *p* < 0.05, ** *p* < 0.01 vs. control.

We evaluated whether ISOR attenuates the expression of genes involved in adipogenesis and mitochondrial function in 3T3-L1 adipocytes. Cells were treated with 0 (control) or 20 µM ISOR, and incubated for 7 days. In ISOR-treated cells, mRNA levels of PPAR-γ, and adipocyte protein 2 (aP2) were significantly lower (34% and 44%, respectively), than those of controls (Figure 4a). Conversely, mRNA levels of PGC-1α, NRF1, and Tfam, which regulate mitochondrial biosynthesis, were significantly higher (2.0-, 2.3-, or 1.9-fold, respectively), in ISOR-treated cells than in controls (Figure 4b). The mRNA level of CPT-1α, which regulates fatty acid oxidation, was also significantly higher by 3.2-fold in ISOR-treated cells, than in control (Figure 4b).

### 2.5. Effect of ISOR on mtDNA Content in Adipocytes

**Figure 5:**
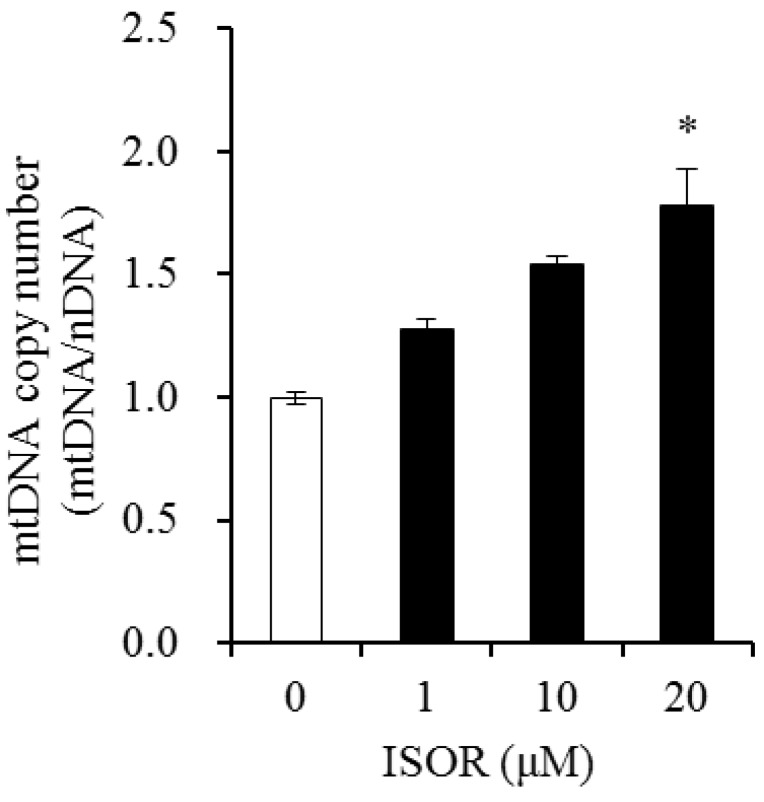
Effects of ISOR on mtDNA content in in adipocytes. 3T3-L1 adipocytes were treated with 0 (control), 1, 10, and 20 μM ISOR, for 7 days. mtDNA copy number was measured by qRT-PCR. Values are expressed as means ± SE (*n* = 3) of three independent experiments. mtDNA, mitochondrial DNA; nDNA, nuclear DNA; ISOR, isorhamnetin. * *p* < 0.05 vs. control.

The effect of ISOR on mtDNA copy number, was determined by the ratio of mtDNA to nuclear DNA (nDNA) in 3T3-L1 adipocytes. Cells were incubated with 0 (control), 1, 10, and 20 μM ISOR, and incubated for 7 days. In the presence of 20 μM ISOR, the mtDNA/nDNA ratio significantly increased 1.78-fold, compared to the control (Figure 5).

### 2.6. Effect of ISOR on AMPK Activity in Adipocytes

**Figure 6:**
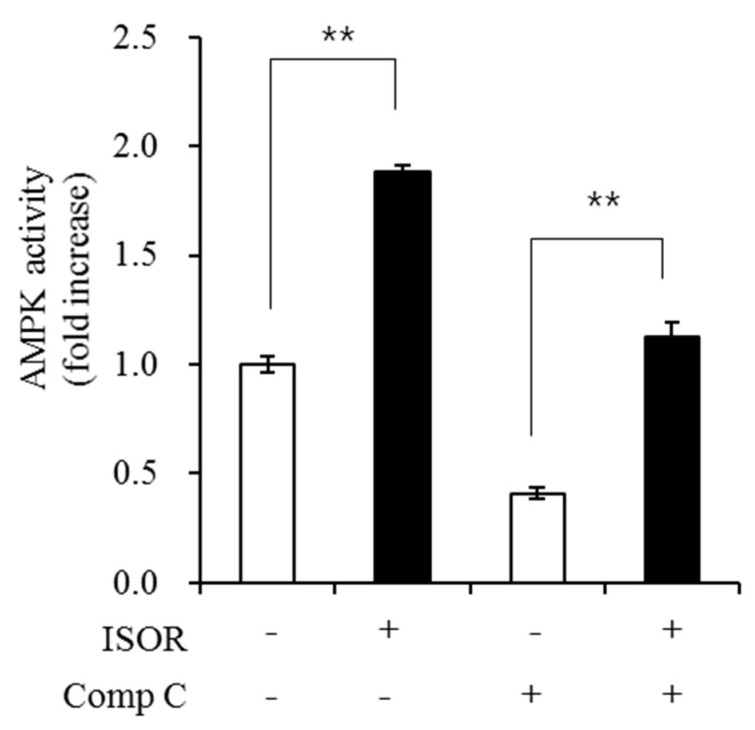
Effect of ISOR on AMPK activity in adipocytes. 3T3-L1 adipocytes were treated with 0 (control) or 20 μM ISOR, for 7 days and incubated with 1 μM of Comp C as an AMPK inhibitor for 16 h. AMPK activity was determined using an AMPK Kinase Assay Kit. Values are expressed as means ± SE (*n* = 3) of three independent experiments. AMPK, AMP-activated protein kinase; ISOR, isorhamnetin; Comp C, compound c. ** *p* < 0.01 vs. control (without or with Comp C.)

To evaluate whether ISOR affected the activity of AMPK, which modulates lipid metabolism and mitochondrial biogenesis in 3T3-L1 adipocytes, cells were treated with 0 (control) or 20 μM ISOR for 7 days and incubated with 1 μM of compound C (Comp C) as an AMPK inhibitor for 16 h. AMPK activity in the presence of 20 μM ISOR. without or with Comp C was significantly higher (at 1.9-, or 2.7-fold, respectively), than those of the controls (Figure 6).

## 3. Discussion

Mitochondria are essential for maintaining metabolic homeostasis in white adipose tissue because, they are involved in adipogenesis, lipogenesis, lipolysis, fatty acid esterification, and glyceroneogenesis [22]. Growing interest in food components that regulate mitochondrial function, has led to the study of several components which reportedly induce mitochondrial biogenesis, such as resveratrol [23], quercetin [13,14], curcumin [24], and epigallocatechin-3-gallate (EGCG) [25]. Specifically, EGCG was reported to increase mitochondrial biogenesis by AMPK activation in brown adipose tissue [25]. This study evaluated the effects of ISOR on mitochondrial biogenesis, and AMPK activation during 3T3-L1 adipocyte differentiation.

Adipogenesis, the process whereby preadipocytes differentiate into adipocytes plays a key role in the development of obesity. Adipocyte differentiation increases triglyceride storage, and GPDH plays an important role in the conversion of glycerol into triglycerides. In the adipocyte differentiation process, PPAR-γ is a principal transcription factor, and aP2 is regulated by PPAR-γ [26]. The 3T3-L1 cell line is a well-established preadipocyte cell line, and a reliable model for studying the conversion of preadipocytes into adipocytes [27]. Particularly, the advantages of this cell line are that it is easy to cultivate, and it provides a homogeneous reaction depending on the experimental conditions [28]. These 3T3-L1 cells have been useful in studying, the regulatory mechanisms during the differentiation process of various food compounds [19,29,30]. This study, investigated the effect of ISOR on adipocyte differentiation using 3T3-L1 cells. In a previous study, ISOR was reported to reduce TG content and GPDH activity, as well as, to downregulate PPAR-γ and CEBP-α genes in 3T3-L1 adipocytes [19]. In agreement with these results we observed that ISOR inhibited, lipid and TG accumulation, and GPDH activity, whilst suppressing PPAR-γ and aP2 gene expression during 3T3-L1 adipocyte differentiation. Thus, our results indicated that ISOR had beneficial anti-adipogenic effects. 

When quercetin was administered to mice, ISOR, a metabolite of quercetin, was detected in high concentrations in the plasma [31]. Quercetin has been reported to improve mitochondrial function in 3T3-L1 cells, and diet-induced obese mice [13,14]. However, it was not clear whether ISOR played a role in mitochondrial function during adipogenesis. To evaluate whether ISOR affects mitochondrial function, this study measured the mRNA levels of mitochondria genes, such as PGC-1α, NRF-1, Tfam, and CPT-1α, during 3T3-L1 adipocyte differentiation. PGC-1α controls mitochondrial biogenesis by enhancing several transcription factors, including NRF-1 and NRF-2 [6,7]. NRF-1 is a potent regulator of the expression of nuclear genes required for mitochondrial function [8]. PGC-1α and NRF-1, coactivate the expression of Tfam, which is a mitochondrial transcription factor that plays an important role in the maintenance of mitochondrial DNA [8,32]. CPT-1α is an enzyme present in the outer mitochondria membrane that regulates beta oxidation by transferring long-chain fatty acids into the mitochondria [33]. We found that ISOR induced the expression of PGC-1α, NRF-1, Tfam, and CPT-1α during 3T3-L1 adipocyte differentiation. Therefore, it could be postulated that the anti-adipogenic effect of ISOR was partially associated with the expression of genes related to mitochondrial biogenesis and beta oxidation.

To investigate the effects of ISOR on mtDNA replication during 3T3-L1 adipocyte differentiation, mtDNA copy number was measured by qPCR. Measuring the mtDNA replication rate is one approach to monitor mitochondrial biogenesis [34]. Resveratrol was shown to increase mtDNA, and the expression of NRF1, Tfam and PGC-1α in endothelial cells [23]. Quercetin increased the expression of genes associated with mitochondrial biogenesis, such as PGC-1α, NRF-1, and Tfam, and mtDNA content in the epididymal adipose tissue and 3T3-L1 cells [13,14]. Curcumin increased the mtDNA copy number and PGC-1α deacetylation in the skeletal muscle of rats [21]. In this study, ISOR increased the mtDNA copy number in a dose-dependent manner during 3T3-L1 adipocyte differentiation, suggesting it may regulate mitochondrial replication.

AMPK plays an important role in regulating intracellular energy metabolism, and has been identified as a major regulator of mitochondrial biogenesis [35]. Active AMPK promotes mitochondrial biogenesis and function through PGC-1α and NRFs [36]. A recent report showed, that ISOR effectively protects against mitochondrial dysfunction and oxidative stress via the activation of the AMPK pathway in HepG2 cells [21]. Our results showed an increase of AMPK activation by ISOR during 3T3-L1 adipocyte differentiation. Thus, it appeared that the mitochondrial biogenic capacity of ISOR involved activation of the AMPK pathway in adipocytes.

**Figure 7:**
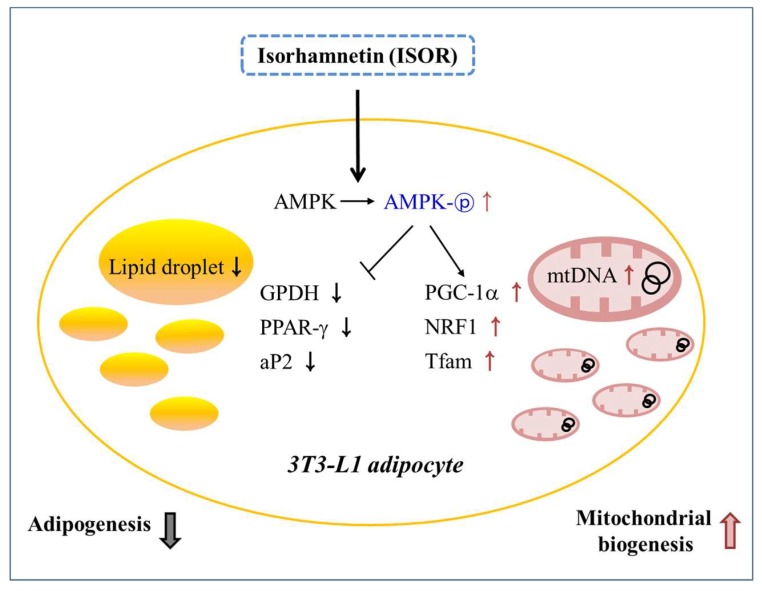
Schematic diagram showing the possible mechanisms of ISOR on adipogenesis, and mitochondrial biogenesis during 3T3-L1 adipocyte differentiation.

In conclusion, the results of the study suggested that ISOR may have beneficial effects on mitochondrial biogenesis during 3T3-L1 adipocyte differentiation. It is likely that ISOR may be related to the stimulation of mitochondrial gene expression and mtDNA replication, and to the activation of AMPK (Figure 7). Therefore, ISOR may be useful as a potential food ingredient to prevent obesity-associated mitochondrial dysfunction.

## 4. Materials and Methods 

### 4.1. Materials

The 3T3-L1 cells were obtained from the American Type Culture Collection (Manassas, VA, USA). Dulbecco’s modified Eagle’s medium (DMEM), glutamine, penicillin-streptomycin, fetal bovine serum (FBS), bovine calf serum (BCS), and TRIzol reagent were obtained from Invitrogen (Carlsbad, CA, USA). The cell count kit-8 (CCK-8) was purchased from Dojindo Laboratories (Kumamoto, Japan). An assay kit for TG was obtained from Asan Pharmaceutical Co. (Seoul, Korea). The GPDH activity assay kit was procured from Takara (Kyoto, Japan). The bicinchoninic acid (BCA) protein assay kit was obtained from Thermo Scientific (Pittsburgh, PA, USA). The Puregene DNA isolation kit was purchased from Qiagen (Chatsworth, CA, USA). M-MLV reverse transcriptase and AccuPower® 2X GreenStarTM qPCR Master Mix were purchased from Bioneer (Daejeon, Korea). The AMPK Kinase Assay kit was purchased from Cyclex (Nagano, Japan) and Isorhamnetin was obtained from Sigma Co. (St Louis, MO, USA)

### 4.2. Cell Culture

**Figure 8:**
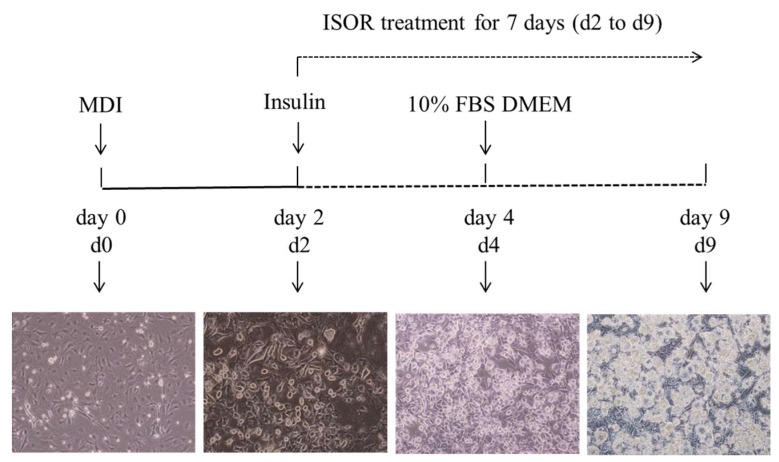
Experiment scheme and pattern of 3T3-L1 preadipocyte differentiation.

The culture of 3T3-L1 cells was performed as described previously [37]. Mouse 3T3-L1 preadipocytes were maintained in DMEM containing 10% (*v*/*v*) BCS, 100 units/mL penicillin, 100 μg/mL streptomycin, and 2 mM glutamine under conditions of 37 °C and 5% CO_2_. For the induction of adipocyte differentiation, the preadipocytes were cultured to confluence (day 0, d0) and were exposed to a differentiation medium containing 0.5 mM isobutylmethylxanthine, 1 μM dexamethasone, and 5 μg/mL insulin (MDI) for 2 days (d2) in DMEM containing 10% FBS. The cells were cultured with 5 μg/mL insulin for 2 days (d4), and then subsequently cultured in DMEM containing 10% FBS for 5 days (d9) (Figure 8). Isorhamnetin (ISOR) was treated with the medium for 7 days (from d2 to d9). Cells incubated without treatment served as a control. All assays were carried out in triplicate. 

### 4.3. Cell Viability Assay 

The cytotoxicity of ISOR in adipocytes was evaluated using the WST-8 [2-(2-methoxy-4-nitropheyl)-3-(4-nitrophenyl)-5-(2,4-dinitrophenyl)-2H-tetrazolium, monosodium salt method, with a commercial CCK-8 kit as described previously [37]. Differentiated 3T3-L1 adipocytes were incubated for 1, 2, 5, or 7 days with ISOR (at a dose of 0, 0.1, 0.5, 1, 10, 20, or 50 µM). The absorbance was read using a Varioskan plate reader (Thermo Electron, Waltham, MA, USA) at 450 nm, and the results are expressed as a percentage of the control. All assays were carried out in triplicate.

### 4.4. Oil-Red O Staining

Lipid content was measured as previously described [37]. Briefly, 3T3-L1 adipocytes were treated with 20 μM of ISOR. On days 2, 5, and 7, the cells were rinsed in phosphate-buffered saline (PBS) (pH 7.4) and then fixed in 10% (*v*/*v*) formalin in PBS. To quantify accumulated lipid levels, cells were stained for 15 min with oil-red O dye (6 parts of saturated oil-red O in isopropanol and 4 parts of water). Oil droplets stained with oil-red O were dissolved in 4% (*v*/*v*) Nonidet P-40 and isopropanol and quantified by measuring absorbance at 520 nm. The results were expressed as a percentage of the control.

### 4.5. TG Assay

For the measurement of intracellular triglyceride (TG), a colorimetric TG Assay kit was used according to the method as described previously [37]. The 3T3-L1 adipocytes were lysed using a buffer containing 1% Triton X-100 in PBS, and the level of TG was determined using a commercial TG assay kit. Cellular TG content was then normalized to the protein concentration and measured by a BCA protein assay kit.

### 4.6. GPDH Activity

Glycerol-3-phosphate dehydrogenase (GPDH) activity was analyzed as described previously [37], using a commercial kit. In brief, 3T3-L1 adipocytes were treated with 0, 1, 10, or 20 μM of ISOR. The cells were then disrupted by homogenization and centrifuged at 4 °C for 10 min. The supernatant was assayed for GPDH activity by monitoring the decrease of NADH in the presence of dihydroxyacetone phosphate and measuring absorbance at 340 nm. Cellular protein level was determined using a BCA protein assay kit. The results were expressed as a percentage of the control.

### 4.7. Quantitative Real-Time PCR

Total RNA was extracted from 3T3-L1 adipocytes using TRIzol Reagent. The cDNAs were synthesized from 4 μg RNA, using an M-MLV reverse transcriptase. Quantitative real-time PCR (qRT-PCR), was then carried out in 25 μL of Universal SYBR^®^ Green PCR Master Mix using a fluorometric thermal cycler (Rotor-GeneTM 2000; Corbett Research, Mortlake, NSW, Australia). Primer3 software (Version 2.3.6, Boston, MA, USA) was used for the primer design [38]. The sequences of the primers used are presented in Table 1. For relative quantification, the delta–delta Ct method was used [39], and β-actin was used as an endogenous control. Values presented represent fold changes compared to the control.

### 4.8. mtDNA Analysis 

The amount of mitochondrial DNA (mtDNA) was determined according to previous work [18]. The genomic DNA was detected in adipocytes via a Puregene DNA isolation kit in accordance with the manufacturer’s instructions. The mtDNA content was assessed using qRT-PCR as the ratio of mitochondrial gene (Cox1, cytochrome oxidase subunit I) to nuclear gene (GAPDH, glyceraldehyde-3-phosphate dehydrogenase).

### 4.9. AMPK Activity Assay

AMP-activated protein kinase (AMPK) activity was performed as described previously [18], using an AMPK Kinase Assay kit in accordance with the manufacturer’s instructions. Samples were briefly incubated for 30 min at 30 °C, on a precoated plate with a substrate peptide that corresponded to mouse IRS-1. AMPK activity was measured by monitoring phosphorylation of Ser 789 in IRS-1, using an anti-mouse phospho-Ser 789 IRS-1 monoclonal antibody and peroxidase-coupled anti-mouse IgG. Conversion of the chromogenic substrate tetramethylbenzidine was quantified by measuring absorbance at 450 nm. Protein was determined using a BCA protein assay kit. Values for AMPK activity were expressed as a fold-increase of the untreated control.

### 4.10. Statistical Analysis 

Values were expressed as mean ± standard error of the mean (SEM). Statistical analyses were performed using SPSS software (version 23; IBM Corporation, Armonk, NY, USA). The significance of differences among same concentration of treatment groups was determined with a Student’s *t*-test (two-tailed). Significant differences among different concentrations of treatment group were analyzed using a one-way analysis of variance (ANOVA), followed by Tukey’s multiple comparison tests. *p* < 0.05 indicated a significant difference.

## Figures and Tables

**Table 1:** Primers used for qPCR.

Name	GeneBank No.	Primer Sequence (5′-3′)
aP2	NM_024406	F: CAAGTGCTCAAGTTTGGCGC
		R: CAAGAACCACCCCGAAGCTC
β-actin	NM_007393	F: GGACCTGACAGACTACCTCA
		R: GTTGCCAATAGTGATGACCT
CPT-1α	NM_013495	F: GTGTTGGAGGTGACAGACTT
		R: CACTTTCTCTTTCCACAAGG
NRF1	NM_010938	F: AAGTATTCCACAGGTCGGGG
		R: TGGTGGCCTGAGTTTGTGTT
PGC-1α	NM_008904	F: GGGCCAAACAGAGAGAGAGG
		R: GTTTCGTTCGACCTGCGTAA
PPAR-γ	NM_011146	F: TTGATTTCTCCAGCATTTCT
		R: TGTTGTAGAGCTGGGTCTTT
Tfam	NM_009360	F: GAGGCCAGTGTGAACCAGTG
		R: GTAGTGCCTGCTGCTCCTGA
